# Milk metabolomics analyses of lactating dairy cows with divergent residual feed intake reveals physiological underpinnings and novel biomarkers

**DOI:** 10.3389/fmolb.2023.1146069

**Published:** 2023-04-04

**Authors:** Dagnachew Hailemariam, Mohsen Hashemiranjbar, Ghader Manafiazar, Paul Stothard, Graham Plastow

**Affiliations:** ^1^ Department of Agricultural, Food, and Nutritional Science, University of Alberta, Edmonton, AB, Canada; ^2^ Animal Science and Aquaculture Department, Faculty of Agriculture, Dalhousie University, Halifax, NS, Canada

**Keywords:** milk metabolome, biomarker, feed efficiency, physiology, dairy cows

## Abstract

The opportunity to select for feed efficient cows has been limited by inability to cost-effectively record individual feed efficiency on an appropriate scale. This study investigated the differences in milk metabolite profiles between high- and low residual feed intake (RFI) categories and identified biomarkers of residual feed intake and models that can be used to predict residual feed intake in lactating Holsteins. Milk metabolomics analyses were undertaken at early, mid and late lactation stages and residual feed intake was calculated in 72 lactating dairy cows. Cows were ranked and grouped into high residual feed intake (RFI >0.5 SD above the mean, *n* = 20) and low residual feed intake (RFI <0.5 SD below the mean, *n* = 20). Milk metabolite profiles were compared between high residual feed intake (least efficient) and low residual feed intake (most efficient) groups. Results indicated that early lactation was predominantly characterized by significantly elevated levels of medium chain acyl carnitines and glycerophospholipids in high residual feed intake cows. Citrate cycle and glycerophospholipid metabolism were the associated pathways enriched with the significantly different metabolites in early lactation. At mid lactation short and medium chain acyl carnitines, glycerophospholipids and amino acids were the main metabolite groups differing according to residual feed intake category. Late lactation was mainly characterized by increased levels of amino acids in high residual feed intake cows. Amino acid metabolism and biosynthesis pathways were enriched for metabolites that differed between residual feed intake groups at the mid and late lactation stages. Receiver operating characteristic curve analysis identified candidate biomarkers: decanoylcarnitine (area under the curve: AUC = 0.81), dodecenoylcarnitine (AUC = 0.81) and phenylalanine (AUC = 0.85) at early, mid and late stages of lactation, respectively. Furthermore, panels of metabolites predicted residual feed intake with validation coefficient of determination (*R* ^2^) of 0.65, 0.37 and 0.60 at early, mid and late lactation stages, respectively. The study sheds light on lactation stage specific metabolic differences between high-residual feed intake and low-residual feed intake lactating dairy cows. Candidate biomarkers that distinguished divergent residual feed intake groups and panels of metabolites that predict individual residual feed intake phenotypes were identified. This result supports the potential of milk metabolites to select for more efficient cows given that traditional residual feed intake phenotyping is costly and difficult to conduct in commercial farms.

## Introduction

Feed cost represents a large proportion of the variable costs of dairy production and it has increased substantially over time (Berry and Crowley, 2013). Selection for feed efficiency has both economic and environmental benefits by addressing the increasing cost of feed and environmental concerns over the carbon footprint of dairy cattle production (Basarab et al., 2013). Residual feed intake (RFI) is a measure of feed efficiency and is defined as the difference between actual and predicted feed intake after accounting for production and maintenance (Koch et al., 1963). Low RFI (the most efficient) cows consume less feed for the same milk output and emit less methane compared to the high RFI (least efficient) cows (Hailemariam et al., 2021). Despite its importance, RFI is difficult and costly to include in an industry wide recording scheme for the purpose of generating accurate estimates for direct selection in the breeding program. Therefore, RFI is designated as a hard-to-measure trait for the dairy industry. Alternatively, low cost and easy-to-measure biomarker models associated with underlying metabolic differences in RFI could provide a potential alternative. Biomarkers offer indirect measurement of traits that would otherwise be expensive to directly measure.

The between-animal differences in RFI could be determined by digestive ability (Potts et al., 2017) ruminal microbial composition (Jewell et al., 2015), feed intake pattern, fermentation and digestion of feed, anabolic and catabolic metabolism, physical activity and thermoregulation (Herd and Arthur, 2009). It has been proposed that variation in RFI may represent inherent variation in basic metabolic processes that determine production efficiency (Cantalapiedra-Hijar et al., 2018). Potts et al. (2017) speculated differences in post absorptive metabolic processes, heat production, or energy utilization for maintenance account for more of the variation in RFI than digestibility.

The mammary gland becomes metabolically active and is an energetically demanding tissue during lactation (Hanigan et al., 2009). Hanigan and Baldwin (1994) constructed a model of energy metabolism in the udder of lactating cows and showed that ATP generated was used for maintenance and synthesis of milk components. Milk components are synthesized in mammary epithelial cells and milk precursors are constantly absorbed from blood (Knight et al., 1994). Proteins, some amino acids, fats and lactose are synthesized and secreted by the mammary epithelial cells (Xiao and Cant, 2005; Rezaei et al., 2016). Branched-chain amino acids are catabolized to support milk production (Li et al., 2009).

Given the vital role of the mammary gland in the energy metabolism of lactating dairy cows, we used a milk metabolomics approach to study the biological mechanisms of divergence in feed efficiency. High-throughput milk metabolomics have been used to study the metabolism (Tian et al., 2016) and pathophysiology (Klein et al., 2011; Sun et al., 2017) of dairy cows. To the best of our knowledge milk metabolomics data had not been used to study the physiology of feed efficiency in dairy cows. In this study, we investigated the differences in milk metabolite profiles between high and low RFI cows at early, mid and late lactation stages. In addition, candidate biomarkers that can distinguish cows between high and low RFI groups and panels of metabolites that can be used to predict individual RFI phenotypes at each stage of lactation were identified.

## Materials and methods

### Animals and diet

**FIGURE 1:**
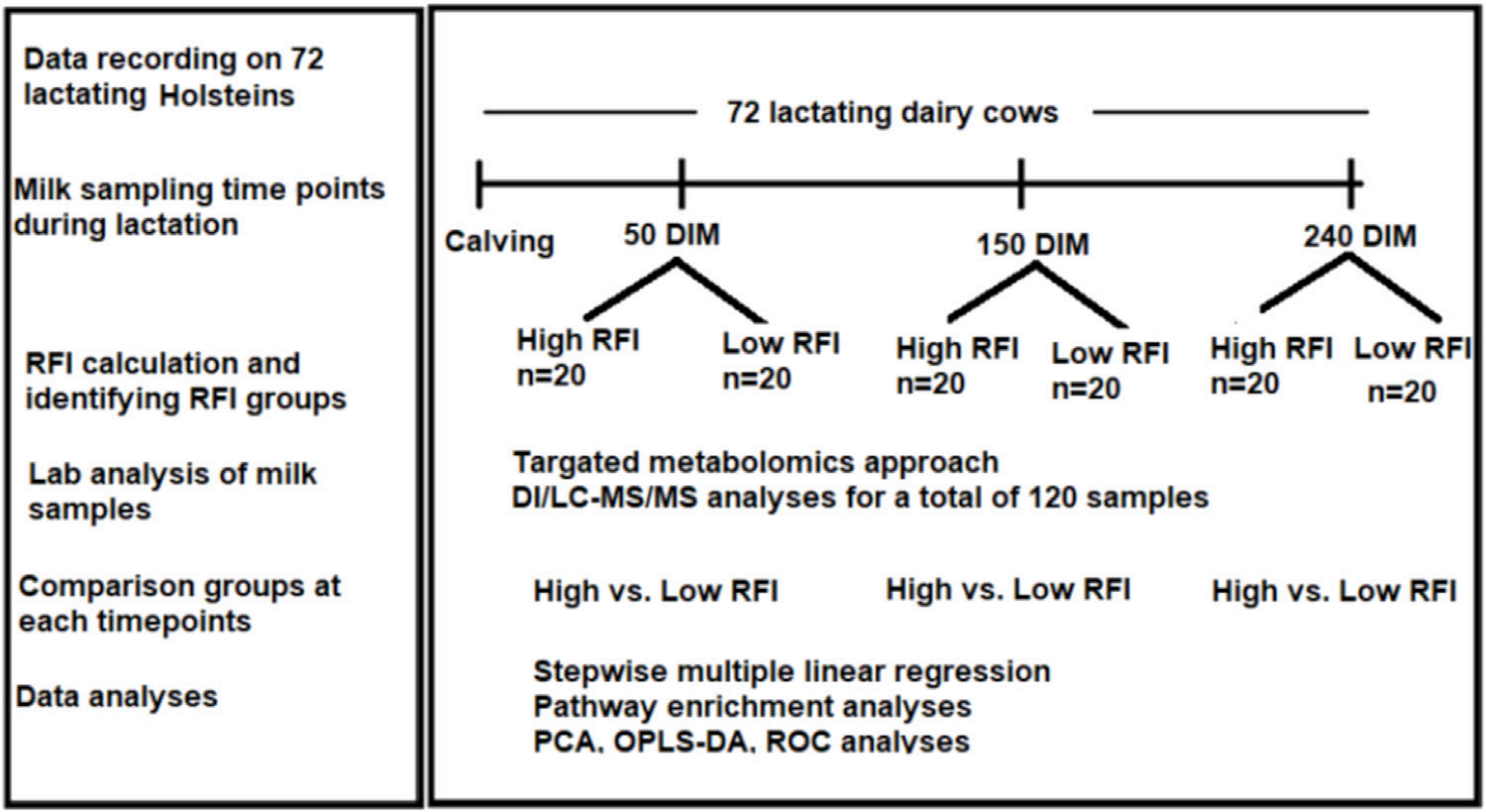
Workflow of the study showing calculation of RFI in 72 cows from 3–240 DIM and milk samples collection at three time points (50, 150, 240 DIM) during lactation. Cows were ranked and the high and low RFI groups were identified. Milk samples from high and low RFI groups in all the three time points were analyzed using DI/LC-MS/MS method.

The experiment was conducted at the University of Alberta, Dairy Research and Technology Center (DRTC) from June 2017 to October 2018. The study was undertaken on mixed parity lactating Holsteins managed in a tie-stall system. The layout of the study is shown in Figure 1. All the experimental procedures for this study were approved by the University of Alberta Animal Policy and Welfare Committee for Livestock (Study ID: AUP00000170), and animals were cared for in accordance with the guidelines of the Canadian Council on Animal Care (2009). We used 72 mixed parity lactating Holstein cows 3–240 days in milk (DIM). Out of the 72 cows that were used in the study, 40 cows (20 least efficient and 20 most efficient) were selected for comparison. Cows that were culled/died due to disease/s or any other reason before last milk sampling point (240 DIM) were excluded from the experiment. Disease incidences in the window of 2 weeks before each sampling dates were considered in the analysis. The number of cows affected by different diseases during the experimental period varied with the sampling points. At 50 DIM, a total of 6 cows (out of 40) were sick and they had lameness (n = 1), milk fever (n = 3), and mastitis (n = 3). Out the 6 sick cows, 1 had both milk fever and mastitis. At 150 DIM, a total of 4 cows had lameness (n = 2), milk fever (n = 1) and mastitis (n = 2). One cow had both lameness and mastitis. At 240 DIM 2 cows had lameness and 2 had mastitis. One of these had both lameness and mastitis. The forty cows (19 primiparous, 21 multiparous) had parity ranging from one to four and all cows with parity >3 were categorized as “3+” during the analyses.

All cows were fed the same diet and daily ration was offered as total mixed ration (TMR) for *ad libitum* intake to allow approximately 5% feed refusals throughout the experiment. All cows were fed once daily in the morning at 08:00 a.m. Individual offered feed weight in the morning and refusal feed weight left on the next morning were recorded daily. Feed composition, including dry matter (%), crude protein (%), and neutral detergent fiber (%), acid detergent fiber (%), and net energy lactation were determined when the TMR ingredients were changed. The ingredients and chemical composition of the TMR is described in Table 1.

**TABLE 1:** Diet ingredients and chemical composition of total mixed ration for the study cows. Average values of ingredients and chemical composition are presented.

Diet ingredients, % (DM basis)	Ration
Alfalfa hay	11.55
Barley silage^a^	35.28
Rolled grain^b^	33.58
Protein supplement	19.59
Chemical composition	
DM, %	50.57
Crude protein, % of DM	17.09
Acid detergent fiber, % of DM	20.71
Neutral detergent fiber, % of DM	32.26
NE lactation, Mcal/kg	1.81

DM, dry matter; NE, net energy for lactation.

^a^ Rolled gran: Corn and barley.

^b^ Protein supplement: 26.61% amino plus (high bypass soy), 26.25% soy bean meal-47%, 25.75% canola meal, 8.15% F 100 Dairy fat, 4% corn distiller 2010, 2.3% limestone, 2% AFA/canola oil, 1.5% SOD, bicarbonate, 1.2% DICAL PHOS-21%, 1% salt, 0.58% MAG OX-56%, 0.4% nutritec-diamond V mills, 0.1% selenium 1,000 mg/kg, 0.1% ruminant TM, pak, 0.05% ADE VIT, PAK-30% and 0.02% biotin 2%-Rovimix H-2.

### Feed intake, milk yield and composition, and body weight recording

Daily feed intake, weekly milk composition and yield and monthly body weight data were collected on 72 lactating dairy cows from 3–240 days in milk (DIM) at DRTC. Feed intake was calculated as the difference between the amount of feed offered and refused for individual cows on a daily basis. Dry matter percentage of the feed was analyzed every week and daily individual dry matter intake (DMI) was calculated as a product of feed intake and dry matter percentage. Cows were milked twice per day and both PM and AM milk samples were collected separately once per week. Milk samples were collected with bar-coded plastic vials. Samples were stored at +4^O^C temporarily and shipped to Lactanet Canada, Edmonton for milk composition analyses. Mid-infrared (MIR) spectrometry (Foss MilkoScan FT6000; Foss Electric A/S, Hillerød, Denmark) was used to determine milk composition (milk fat, protein and lactose). Milk yield (for the dates of milk sampling) data was obtained from the DRTC where it is recorded as a routine farm activity. Animals were weighed once per month at 7:00 a.m. after milking using Myscale Pro-W810 scale (Gallagher, Canley, United Kingdom).

### Milk sample collection for DI/LC-MS/MS analyses

Milk samples were collected from 72 mixed parity lactating cows at DRTC. Sampling was undertaken 03:00 to 06:00 a.m. before feeding at 3 time points during the lactation period (50, 150 and 240 DIM) and hereafter named as early, mid and late lactation stages, respectively. All the milk samples were collected using a 50 mL tube and temporarily maintained on ice. Following homogenization of the milk sample in each 50 mL tube, aliquots of 500 µL were prepared and stored at −80^O^C until DI/LC-MS/MS analyses.

### Trait derivation for RFI calculation

Metabolic body weight and empty body weight were derived from body weight data recorded once per month. Similarly, milk production energy requirement was derived from milk yield, fat, protein and lactose percentages that were recorded weekly. The daily values of metabolic body weight and empty body weight were predicted from monthly values and daily values of milk production energy requirement were predicted from weekly values using a random regression model. The details of the Legendre polynomial random regression model and calculations of the parameters were described in Manafiazar et al. (2013). After predicting daily values of metabolic body weight from monthly values, empty body weight change was calculated as a difference in empty body weight between two consecutive days (days after minus day before, e.g., empty body weight at fourth -third DIM) from 3–240 DIM. Cows that lose weight after calving had negative empty body weight change values while cows that gain weight had positive values. Empty body weight change was calculated to account for the body tissue mobilization in the RFI calculation during the study period (3–240 DIM). A multiple linear and quadratic regression model was used to predict expected energy intake values from 3–240 DIM.

### Calculation of residual feed intake

Residual feed intake values were calculated for 72 lactating Holsteins as the difference between the actual and expected net energy intake as described in our previous study (Hailemariam et al., 2021). In short, daily actual energy intake, metabolic body weight, empty body weight, empty body weight change and milk production net energy requirements were used to calculate RFI. Mixed parity cows were used in the study and parity was included in the RFI calculation model. The daily average lactation RFI for each individual over 237 days (3—240 DIM) was obtained by dividing the total lactation RFI by the number of days recorded for each cow. Then, the 72 RFI predicted cows were ranked and categorized into most efficient (low RFI: RFI <0.5 SD from the mean) and least efficient (high RFI: RFI >0.5 SD from the mean) at all the three time points (50, 150, 240 DIM) using the same average (3–240 DIM) RFI values.

### Targeted milk metabolomics using TMIC prime assay DI/LC-MS/MS method

All the milk samples were analyzed at The Metabolomics Innovation Center (TMIC, University of Alberta, Edmonton, AB) using a TMIC prime custom assay (DI/LC-MS/MS). A targeted quantitative metabolomics approach was used to analyze the samples using a combination of direct injection mass spectrometry with a reverse-phase LC-MS/MS custom assay. This custom assay, in combination with an ABI 4000 Q-Trap (Applied Biosystems/MDS Sciex) mass spectrometer, was used for the targeted identification and quantification of up to 143 different endogenous metabolites including amino acids, acylcarnitines, organic acids, biogenic amines and derivatives, uremic toxins, glycerophospholipids, sphingolipids and sugars. The method combines the derivatization and extraction of analytes and selective mass-spectrometric detection using multiple reaction monitoring (MRM) pairs. Isotope-labeled and other internal standards were used for metabolite quantification. The custom assay contains a 96 deep-well plate with a filter plate attached with sealing tape and reagents and solvents used to prepare the plate assay. The first 14 wells were used for one blank, three zero samples, seven standards and three quality control samples. For all metabolites except organic acids, samples were thawed on ice and were vortexed and centrifuged at ×13,000 g. Ten µL of each sample was loaded onto the center of the filter on the upper 96-well plate and dried in a stream of nitrogen. Subsequently, phenyl-isothiocyanate was added for derivatization. After incubation, the filter spots were dried again using an evaporator. Extraction of the metabolites was then achieved by adding 300 µL of extraction solvent. The extracts were obtained by centrifugation into the lower 96-deep well plate, followed by a dilution step with MS running solvent.

For organic acid analysis, 150 µL of ice-cold methanol and 10 µL of isotope-labeled internal standard mixture was added to 50 µL of milk sample for overnight protein precipitation. After centrifugation at 13,000 g for 20 min 50 µL of supernatant was loaded into the center of wells of a 96-deep well plate, followed by the addition of 3-nitrophenylhydrazine (NPH) reagent. After incubation for 2 h, BHT stabilizer and water were added before LC-MS injection. Mass spectrometric analysis was performed on an API4000 Qtrap^®^ tandem mass spectrometry instrument (Applied Biosystems/MDS Analytical Technologies, Foster City, CA) equipped with an Agilent 1,100 series HPLC system (Agilent Technologies, Palo Alto, CA). The samples were delivered to the mass spectrometer by a liquid chromatography (LC) method followed by direct injection (DI). Data analysis was done using Analyst 1.6.2.

### Statistical analyses

A step wise multiple linear regression analysis in R was used to analyse the differences of milk metabolites between low and high RFI groups. The model was fitted for each metabolite concentration as dependant variable and RFI groups (R), parity (P), age at first calving (AFC), health status (HS), and month and year of sampling (MY) as independent variables. A total of 118 metabolites were analysed using the following model:

$$Y_{ijklmn}=\mu +R_{i}+P_{j}+{MY}_{k}+{AFC}_{l}+{HS}_{m}+{℮}_{ijklmn},$$

where Yijklmn is the metabolite concentration for the *n*th cow tested from the *i*th RFI group (low and high RFI) and *j*th parity (1–3+), *k*th MY, l^th^AFC, *m*th HS and eijklm is the deviation due to the ijklmth cow or error term.

Each metabolite was tested for normal distribution and where appropriate, for data that were not normally distributed, the natural log-transformation was used and back-transformed results are presented. The results were presented as least squares means ± standard error of mean per RFI category. Benjamini Hochberg False Discovery Rate (FDR) method was used to correct the raw *p*-values for multiple comparisons. A threshold of *p* < 0.05 for raw *p*-values and FDR <0.1 were used to identify statistically significant changes in metabolite concentration between RFI groups. Tendencies were declared at 0.05 ≤ *p* < 0.1 for raw *p*-values.

The disease incidences were not equally distributed during the lactation period or in RFI groups. Because of this we coded cows as sick and healthy and included this factor in the model. The concentration of milk metabolites at all the three time points were adjusted for significant fixed effects including health status. To avoid potential bias for the analyses other than the regression, we used metabolome data adjusted for significant fixed effects. Therefore, in all the analyses the effect of disease or other fixed effects on milk metabolite concentration has been taken care of.

Multiple linear regression analyses were used to predict average RFI phenotypes from milk metabolite profiles at early, mid and late lactation stages. Only the metabolites that were significantly different and tended to differ between high vs. low RFI comparisons were fitted in the model. Leave-one-out cross validation (LOOCV) of linear models were done using caret package (Kuhn, 2005).

Metabolite pathway analyses were performed using MetaboAanalyst (Xia et al., 2013) to identify pathways that were enriched for the metabolites that had differential concentration between high vs. low RFI comparison groups. A hypergeometric test was used for over representation analysis and relative–betweenness centrality for pathway topology analysis. *Homo sapiens* was used as a reference with Human Metabolome Database (HMDB) ID since a high proportion of metabolites affected by RFI at early stage of lactation were missing from the bovine metabolome database (BMDB).

Multivariate analyses were performed using MetaboAnalyst (Xia et al., 2013). Principal component analysis (PCA) were used to visualize the change in milk metabolite profiles with lactation stage. Orthogonal partial least squares discriminant analysis (OPLS-DA) was used to assess whether the high and low RFI groups cluster separately on the basis of milk metabolite profiles. To minimize the possibility that any observed separation for an OPLS-DA plot was due to chance, permutation testing involving repeated (2,000 times) OPLS-DA calculations using different random labeling of the samples was performed. A *p*-value (<0.05) was considered significant for the separation observed between the two groups. Metabolites with VIP score >1 were considered as the metabolite significantly contributed for separation of RFI groups. Biomarker profiles and the quality of the biomarkers were determined using receiver-operator characteristic (ROC) curves. A receiver operator characteristic curve shows the sensitivity and specificity of the test and often summarized into a single metric known as the area under the curve (AUC). A rough guide for assessing the utility of a biomarker based on its AUC is 0.9–1.0 = excellent; 0.8 to 0.9 = good; 0.7 to 0.8 = fair; 0.6 to 0.7 = poor; 0.5 to 0.6 = fail (Hailemariam et al., 2014). For this study, we presented the top five metabolites (on the basis of AUC values) at early, mid and late lactation stages.

## Results

### Dynamics of milk metabolites during lactation

**FIGURE 2:**
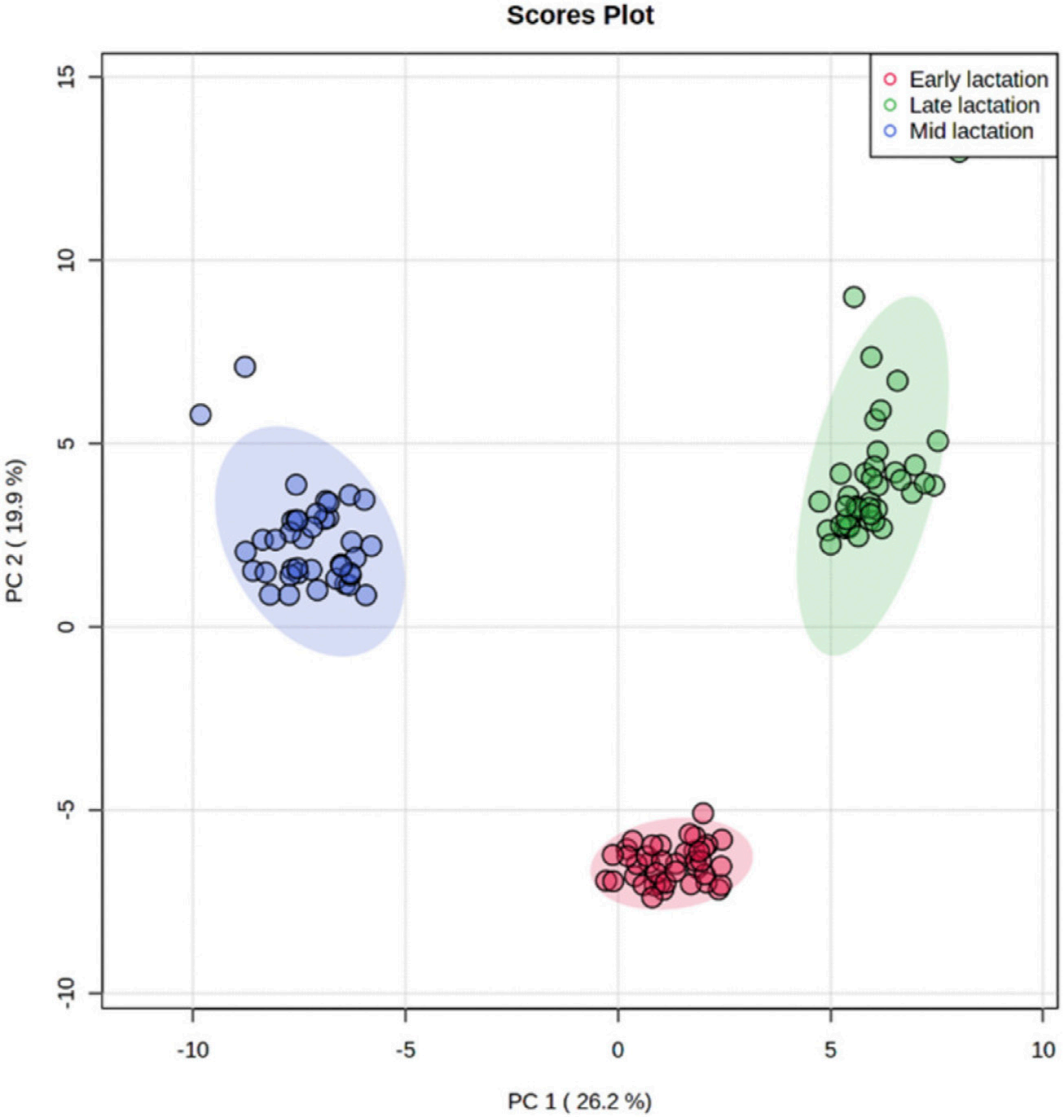
Principal component analysis showing a clear separation in the milk metabolite profiles of cows among early, mid and late lactation stages.

Using targeted DI/LC-MS/MS, 118 milk metabolites were identified and quantified at each of the lactation stages (early, mid and late). To obtain a global perspective on metabolic changes during the lactation period, principal component analysis (PCA) of milk metabolites profiled at early, mid and late lactations were performed without considering RFI grouping. The first two principal components (PCs) accounted for 46.1% of the total variation among samples (Figure 2). The three stages of lactation were clearly distinguished from one another based on the top 2 PCs. Since the three lactation stages strikingly clustered in a non-overlapping manner, we performed the high vs. low RFI comparison independently for each lactation stage.

Next, we compared the high (n = 20) vs. low (n = 20) RFI groups for all 118 metabolites at each lactation stage. In the comparisons of each metabolite concentration between high and low RFI groups, fixed effects such as parity (P), age at first calving (AFC), sampling month and year (MY), health status (HS) were adjusted whenever significant (*p* < 0.05). The result indicated that different sets of metabolites differed (*p* < 0.05) between high-and low RFI groups at each lactation stage. At early lactation, the concentrations of 4 acyl carnitines, 7 glycerophospholipids, 3 biogenic amines, 1 organic acid and betaine were significantly (*p* < 0.05) different between the high and low RFI groups (Table 2). A similar comparison at mid lactation (high vs. low RFI) revealed the differential (*p* < 0.05) concentration of 7 acyl carnitines, 4 amino acids, 7 glycerophospholipids and glucose between the comparison groups (Table 3). The late lactation stage was characterized by differential (*p* < 0.05) concentration of 14 amino acids, 2 biogenic amines, 6 glycerophopholipids, 2 acyl carnitines, 3 organic acids, carnosine and betaine between high and low RFI cows (Table 4).

**TABLE 2:** List of milk metabolites with altered concentrations in the high vs. low RFI comparisons at early lactation stage. The metabolite concentrations were described in least square means (LSM) and corresponding standard error of mean (SEM) in each group with *p*-values and false discovery rate (FDR).

Metabolite (µM)	High RFI (n = 20)	Low RFI (n = 20)	*p*-value	FDR
Decenoylcarnitine (C10:1)	0.13 ± 0.01	0.10 ± 0.01	0.001	0.024
lysoPC a C20:4	0.12 ± 0.02	0.04 ± 0.01	0.001	0.012
lysoPC a C18:1	3.30 ± 0.22	2.12 ± 0.25	0.001	0.008
lysoPC a C18:2	1.40 ± 0.16	0.61 ± 0.23	0.003	0.018
PC aa C40:1	0.05 ± 0.00	0.03 ± 0.01	0.004	0.019
Decadienylcarnitine (C10:20)	0.01 ± 0.00	0.008 ± 0.00	0.008	0.032
Decanoylcarnitine (C10)	0.05 ± 0.00	0.04 ± 0.00	0.009	0.031
Hydroxyhexadecenoylcarnitine (C16:1-OH)	0.13 ± 0.01	0.11 ± 0.01	0.009	0.027
lysoPC a C16:1	0.40 ± 0.03	0.28 ± 0.03	0.009	0.024
lysoPC a C18:0	0.99 ± 0.06	0.75 ± 0.06	0.010	0.024
Total dimethyl arginine	0.55 ± 0.05	0.72 ± 0.05	0.013	0.028
Spermidine	0.45 ± 0.03	0.37 ± 0.02	0.019	0.038
lysoPC a C28:1	0.30 ± 0.03	0.20 ± 0.04	0.021	0.039
Butyric acid	28.8 ± 2.32	21.4 ± 2.26	0.023	0.039
Acetylornithine	0.27 ± 0.05	0.47 ± 0.09	0.027	0.043
Betaine	36.7 ± 3.96	26.5 ± 3.72	0.040	0.060
lysoPC a C26:0	0.16 ± 0.02	0.22 ± 0.02	0.052	0.073
Citric acid	3,897 ± 127	4,271 ± 175	0.060	0.080
Fumaric acid	23.4 ± 1.56	19.5 ± 1.30	0.060	0.076
Butenylcarnitine (C4:1)	0.04 ± 0.00	0.031 ± 0.00	0.070	0.084
SM C16:0	16.1 ± 0.75	14.1 ± 0.75	0.070	0.080
lysoPC a C16:0	5.21 ± 0.32	4.44 ± 0.39	0.082	0.089
PC aa C38:6	0.07 ± 0.01	0.05 ± 0.01	0.084	0.088
Methylmalonic acid	0.13 ± 0.02	0.10 ± 0.01	0.091	0.091

**TABLE 3:** List of milk metabolites with altered concentrations in the high vs. low RFI comparisons at mid lactation stage. The metabolite concentrations were described in least square means (LSM) and corresponding standard error of mean (SEM) in each group with *p*-values and false discovery rate (FDR).

Metabolites (µM)	High RFI (n = 20)	Low RFI (n = 20)	*p*-value	FDR
Dodecenoylcarnitine (C12:1)	0.40 ± 0.02	0.50 ± 0.02	0.001	0.028	
PC aa C36:6	0.03 ± 0.00	0.02 ± 0.00	0.002	0.028	
Methylglutarylcarnitine (C5-M-DC)	0.07 ± 0.00	0.08 ± 0.00	0.003	0.028	
Hydroxypropionylcarnitine (C3-OH)	0.04 ± 0.00	0.05 ± 0.00	0.004	0.028	
LysoPC a C28:1	0.40 ± 0.03	0.30 ± 0.03	0.004	0.022	
Hydroxyhexadecenoylcarnitine (C16:1-OH)	0.02 ± 0.00	0.03 ± 0.00	0.005	0.023	
PC aa C38:6	0.09 ± 0.01	0.06 ± 0.00	0.005	0.020	
Methionine	1.90 ± 0.19	1.20 ± 0.12	0.006	0.021	
Histidine	2.70 ± 0.53	1.20 ± 0.24	0.006	0.019	
Dodecanoylcarnitine (C12)	4.70 ± 0.29	5.70 ± 0.30	0.008	0.022	
Glucose	33,823 ± 1,136	38,742 ± 1,301	0.008	0.020	
PC aa C32:2	0.90 ± 0.07	0.70 ± 0.08	0.019	0.044	
Tryptophan	0.60 ± 0.11	0.40 + 0.06	0.029	0.063	
PC ae C40:6	0.02 ± 0.00	0.03 ± 0.00	0.032	0.064	
Pimelylcarnitine (C7-DC)	0.05 ± 0.00	0.08 ± 0.01	0.033	0.062	
Valine	6.00 ± 1.20	3.30 ± 0.65	0.035	0.061	
PC ae C36:0	0.60 ± 0.04	0.50 ± 0.03	0.039	0.064	
Hexenoylcarnitine (C6:1)	0.05 ± 0.00	0.06 ± 0.00	0.044	0.068	
SM C20:2	0.10 ± 0.00	0.08 ± 0.00	0.048	0.071	
SM C16:0	23.2 ± 1.31	19.6 ± 1.31	0.055	0.077	
SM C18:1	0.74 ± 0.05	0.62 ± 0.04	0.06	0.080	
Arginine	11.5 ± 1.16	8.50 ± 0.89	0.066	0.084	
Creatine	460 ± 36.3	556 ± 36.3	0.068	0.083	
Methylmalonic acid	0.12 ± 0.00	0.10 ± 0.00	0.071	0.083	
Phenylalanine	3.10 ± 0.32	2.40 ± 0.29	0.073	0.082	
LysoPC a C26:1	0.08 ± 0.00	0.05 ± 0.00	0.073	0.079	
SM C16:1	0.70 ± 0.05	0.60 ± 0.04	0.074	0.077	
Creatinine	66.1 ± 3.19	76.5 ± 4.47	0.081	0.081	

**TABLE 4:** List of milk metabolites with altered concentrations in the high vs. low RFI comparisons at late lactation stage. The metabolite concentrations were described in least square means (LSM) and corresponding standard error of mean (SEM) in each group with *p*-values and false discovery rate (FDR).

Metabolites (µM)	High RFI (n = 20)	Low RFI (n = 20)	*p*-value	FDR
Valine	12.0 ± 2.80	3.40 ± 0.79	0.001	0.037
Acetyl ornithine	0.61 ± 0.12	0.20 ± 0.03	0.001	0.019
Lysine	51.8 ± 11.7	18.6 ± 4.89	0.002	0.025
Histidine	25.1 ± 2.19	15.7 ± 2.25	0.002	0.019
Methylhistidine	1.10 ± 0.23	0.40 ± 0.08	0.002	0.015
Tryptophan	1.00 ± 0.21	0.40 ± 0.08	0.003	0.019
Proline	33.8 ± 5.94	16.1 ± 2.59	0.003	0.016
Citrulline	3.30 ± 0.75	1.20 ± 0.27	0.003	0.014
Ornithine	5.90 ± 0.94	3.00 ± 0.44	0.003	0.012
Arginine	15.5 ± 2.29	8.00 ± 1.18	0.003	0.011
Phenylalanine	4.20 ± 0.63	2.20 ± 0.33	0.004	0.013
Serine	9.30 ± 1.17	5.40 ± 0.68	0.005	0.015
Hexadecanoylcarnitine (C16)	0.04 ± 0.01	0.02 ± 0.00	0.006	0.017
Lactic acid	267 ± 66	104 ± 23.7	0.007	0.019
Methionine	2.50 ± 0.35	1.40 ± 0.20	0.01	0.025
Alanine	19.4 ± 7.30	5.20 ± 1.78	0.011	0.025
Total dimethylarginine	0.60 ± 0.06	0.40 ± 0.05	0.011	0.024
PC acyl-alkyl (ae) C40:6	0.03 ± 0.00	0.02 ± 0.00	0.013	0.027
Betaine	123 ± 19.50	80.8 ± 15.9	0.015	0.029
Carnosine	1.00 ± 0.19	0.50 ± 0.09	0.017	0.031
PC diacyl (aa) C36:6	0.03 ± 0.00	0.04 ± 0.00	0.017	0.030
Glutamine	4.12 ± 0.63	2.80 ± 0.53	0.018	0.030
SM C20:2	0.10 ± 0.01	0.07 ± 0.01	0.021	0.034
Pyruvic acid	29.7 ± 3.29	21.1 ± 2.13	0.023	0.035
SM C16:0	23.7 ± 1.65	19.5 ± 1.86	0.025	0.037
PC aa C38:6	0.10 ± 0.01	0.06 ± 0.01	0.032	0.046
Dodecanoylcarnitine (C12)	4.50 ± 0.32	5.40 ± 0.45	0.032	0.044
Choline	353 ± 26.2	276 ± 30.8	0.037	0.049
LysoPC a C18:2	1.80 ± 0.41	1.30 ± 0.35	0.044	0.056
Threonine	6.60 ± 1.51	3.60 ± 0.76	0.05	0.062
Spemidine	0.50 ± 0.07	0.30 ± 0.10	0.052	0.062
Carnitine (C0)	31.6 ± 3.60	21.4 ± 4.39	0.058	0.067
Hexadecadienylcarnitine (C16:2)	0.02 ± 0.00	0.02 ± 0.00	0.063	0.071
Glycine	45.5 ± 10.5	26.1 ± 5.51	0.071	0.077
PC ae C36:0	0.80 ± 0.05	0.60 ± 0.05	0.073	0.077
Creatinine	86.3 ± 3.79	93.8 ± 4.27	0.081	0.083
Hexenoylcarnitine (C6:1)	0.05 ± 0.00	0.06 ± 0.00	0.097	0.097

Notably, acyl carnitines, glycerophospholipids and amino acids were predominantly different (*p* < 0.05) between RFI comparison groups (high vs. low). The concentrations of medium and long chain acyl carnitines (C10:2, C10:1, C10, C16:1-OH) were increased at early lactation stage in the high RFI group, however at the mid lactation stage the concentrations of short chain acyl carnitines (C3-OH, C5-M-DC, C6:1, C7-DC) were significantly decreased in high RFI cows. At the late lactation stage, the levels of a medium (C12) and long chain (C16) acyl carnitines were affected by the RFI grouping and C12 was decreased whereas C16 was increased in high RFI cows.

The comparison of glycerophospholipids (high vs. low RFI) at early lactation stage showed that lysophosphatidylcholines (lysoPC a C20:4, lysoPC a C18:1, lysoPC a C18:2, lysoPC a C16:1, lysoPC a C18:0 and lysoPC a C28:1) were significantly elevated in high RFI cows. Lysophosphatidylcholines are formed by hydrolysis of phosphatidylcholines by lipoprotein-associated phospholipase A2. Interestingly, during mid lactation, phosphatidylcholines (PC aa C38:6, PC aa C32:2, PC aa C36:6 and PC ae C36:0) were elevated in high RFI cows. On the contrary, PC ae C40:6 was significantly decreased in high RFI cows. Among other species of glycerophospholipids, only lysoPC a C28:1 and SM C20:2 were significantly increased in high RFI cows at mid lactation stage. The increased (*p* < 0.05) level of PC aa C38:6 in high RFI cows persisted during the late lactation stage. The levels of PC ae C40:6 and PC aa C38:6 were elevated in high RFI cows whereas PC aa C36:6 decreased in high RFI cows at the late lactation stage. In addition, SM C16:0, SM C20:2 and lysoPC a C18:2 were significantly increased in high RFI cows.

The high and low RFI cows did not differ (*p* > 0.05) in any of the amino acids compared at the early stage of lactation. At the mid lactation stage, however, valine, tryptophan, methionine and histidine were significantly elevated in high RFI cows. Interestingly, the late lactation stage was characterized by significant elevation of amino acids: valine, lysine, histidine, methylhistidine, tryptophan, proline, citrulline, ornithine, arginine, phenylalanine, serine, methionine, alanine, glutamine, threonine and glycine. Valine, one of the branched chain amino acids, was markedly decreased in low RFI cows (Table 4). Among the biogenic amines, total dimethyl arginine and acetyl ornithine were significantly decreased in high RFI cows, whereas spermidine was significantly increased in the high RFI group at the early stage of lactation. Acetyl ornithine and total dimethylarginine were also significantly increased in high RFI cows at the late lactation stage. The comparison of organic acids (high vs. low RFI) at the early lactation stage revealed that the level of butyric acid was elevated in high RFI cows. In addition, fumaric and methyl malonic acid tended to increase in high RFI cows, while citric acid showed a tendency to increase in low RFI. Methyl malonic acid maintained a similar trend in mid lactation, where tendency of increase was observed in high RFI groups. At the late lactation stage, however, pyruvic and lactic acid significantly increased in high RFI cows (Table 4). The high RFI cows had consistently elevated levels of betaine at early and late lactation stages.

**FIGURE 3:**
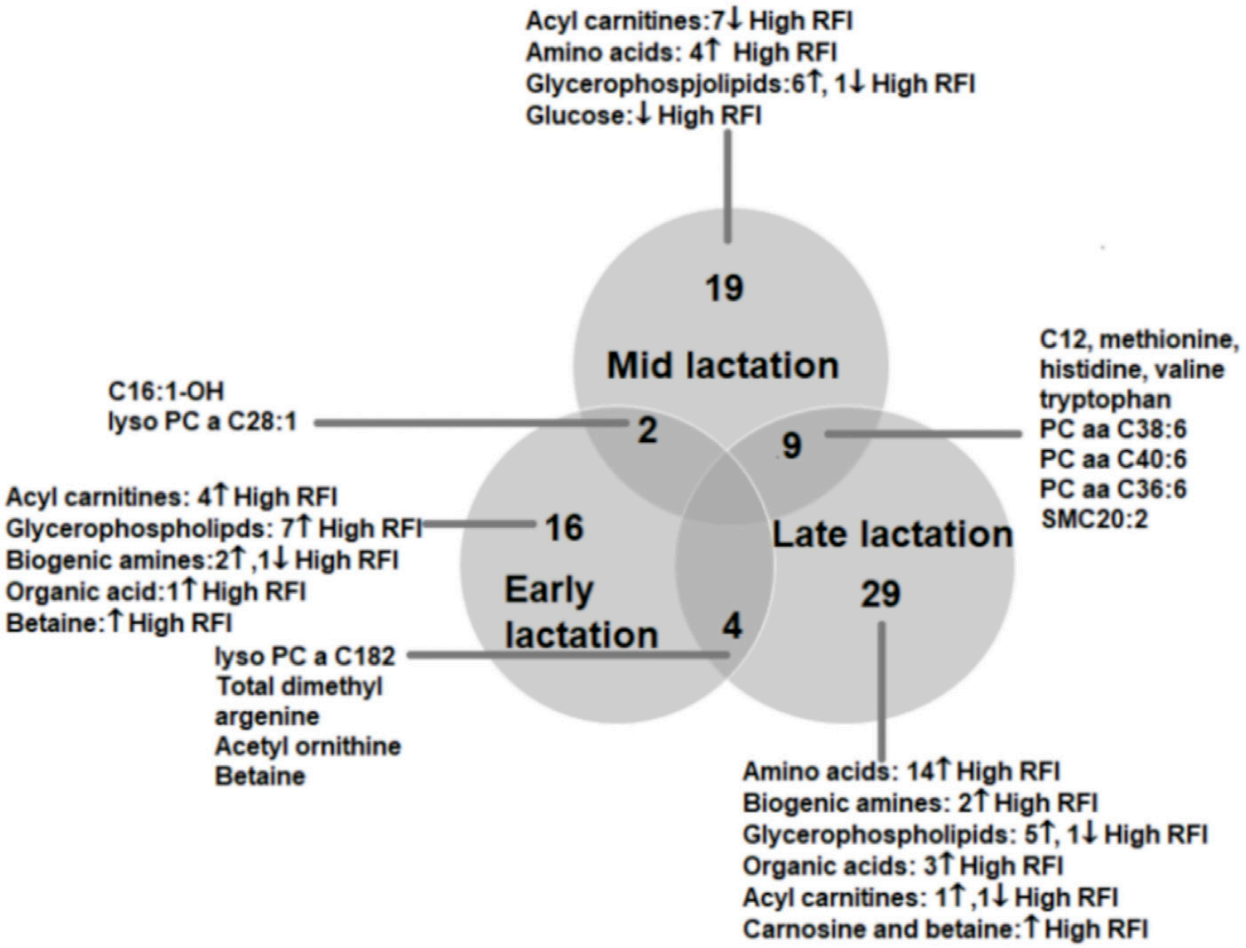
Venn diagram showing the number of metabolites altered between high and low RFI groups at early, mid and late lactation stages. The number of commonly altered metabolites between lactation stages are shown at the intersections of the circles representing the different lactation stages.

To understand the change in the types of metabolites affected by RFI grouping, we compared the significantly different metabolites between the lactation stages (early vs. mid, early vs. late and mid vs. late). The early vs. mid-lactation comparison showed that C16:1-OH, lysoPC a 28:1, SM C16:0, PC aa 38:6 and methyl malonic acid consistently differed between the RFI groups. In a comparison between early and late stages of lactation, the concentrations of lysoPC a C18:2, PC aa C38:6, SMC16:0, total dimethyl arginine, spermidine, acetylornithine and betaine were commonly altered while, C12, C16:1, methionine, histidine, arginine, phenylalanine, valine, tryptophan, PC aa C38:6, PC aa C36:6, PC ae C36:0, SMC20:2, SMC16:0 and creatinine were commonly different in the mid vs. late comparison (Figure 3). The mid and late lactation stage had more common metabolites than early and mid or -late lactation stage.

### Enriched pathway shift with lactation stages

**FIGURE 4:**
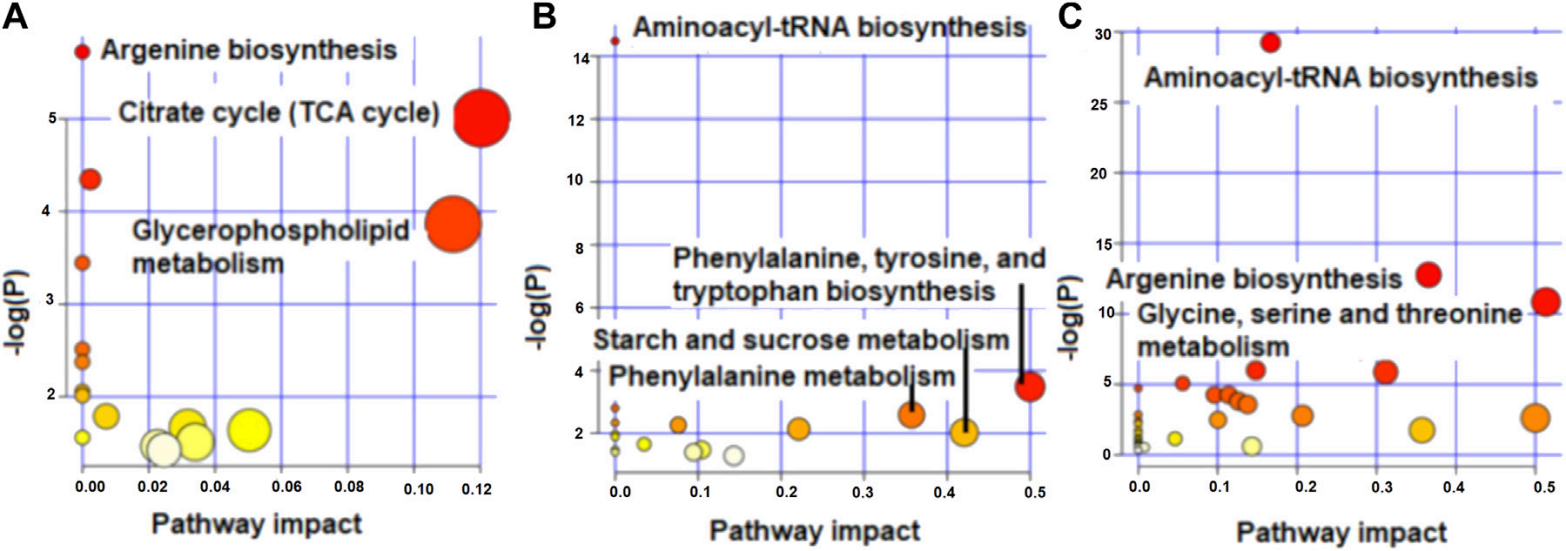
Metabolome view map showing enriched pathways for the metabolites that differed in concentration between high and low RFI groups at early **(A)**, mid **(B)** and late **(C)** stages of lactation.

The enriched pathways (*p* < 0.05) for the metabolites that differed and tended to differ in concentration between high and low RFI cows at early, late and mid lactation are shown in Figure 4. Citrate cycle (TCA cycle) and glycerophospholipid metabolism had a pathway impact value higher than 0.1, which is the cutoff value for relevance (Figure 4A) at early lactation stage. Phenylalanine, tyrosine and tryptophan biosynthesis and phenylalanine metabolism were enriched pathways at mid lactation. Among the significant pathways, only phenylalanine metabolism and phenylalanine, tyrosine and tryptophan biosynthesis had a pathway impact factor greater than 0.1 (Figure 4B). At late lactation stage, aminoacyl-tRNA biosynthesis, argenine biosynthesis, glycine, serine and threonine metabolism were the top three pathways significantly (*p* < 0.05) enriched between high and low RFI groups (Figure 4C).

### Multivariate analyses of milk metabolites during lactation

**FIGURE 5:**
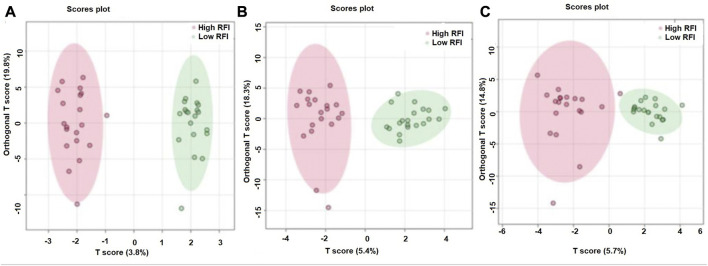
Orthogonal partial least squares discriminant analysis showing a clear separation between the least and most efficient cows at early **(A)**, mid **(B)** and late **(C)** lactation stages.

**FIGURE 6:**
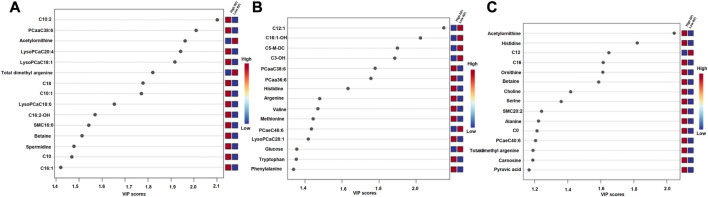
Metabolites ranked by variable importance in projection (VIP) at the early **(A)**, mid **(B)** and late **(C)** lactation stages. The VIP score plots indicate the top 15 metabolites that distinguished high and low RFI groups at each of the three time points.

The supervised orthogonal partial least squares discriminant analysis (OPLS-DA) approach was used to explore the clustering of milk samples from high and low RFI cows at early (Figure 5A), mid (Figure 5B) and late (Figure 5C) lactations. The OPLS-DA score plot showed a clear separation of the high and low RFI cows at early (*Q* ^2^ = 0.68, *p* < 0.001; *R* ^2^Y = 0.99, *p* = 0.008), mid (*Q* ^2^ = 0.65, *p* < 0.001; *R* ^2^ Y = 0.98, *p* < 0.001) and late (*Q* ^2^ = 0.63, *p* < 0.001; *R* ^2^Y = 0.88, *p* < 0.001) lactation. The variable importance in projection (VIP) analyses at early, mid and late lactation stages showed the top 15 metabolites that contributed to the separation of high and low RFI groups were shown in Figures 6A–C, respectively. The top 15 metabolites at all the three time points had VIP >1. The step wise linear regression analysis (metabolites significantly different between RFI groups) and VIP (metabolites with VIP >1) identified similar metabolites. Out of the top 15 metabolites (VIP) that contributed to the separation of high and low RFI groups, 12, 13, 14 metabolites were significantly different between RFI groups at early, mid and late lactation, respectively.

A ROC analyses was undertaken to identify the metabolites that could classify cows into high or low RFI categories. Candidate biomarkers were ranked based on the area under the curve (AUC) and the top five candidate biomarkers at each of the three stages of lactation are shown in Table 5. At the early stage of lactation, C10, acyl ornithine, SM C16:0, lysoPC a C16:1 and lysoPC a C18:1 were the top five candidate biomarkers with AUC values of 0.81, 0.75, 0.75, 0.74 and 0.74, respectively. Dodecenoylcarnitine (C12:1), histidine, creatinine, C3:1, C16:1OH were the top five metabolites that were able to distinguish cows in the high or low RFI categories. During the late stage of lactation, phenylalanine, valine, methionine, arginine, acetyl ornithine were the top 5 metabolites with AUC values of 0.85, 0.81, 0.79, 0.79 and 0.78, respectively (Table 5).

**TABLE 5:** Top 5 metabolites (biomarkers) with corresponding AUC and CI at early, mid and late lactation stages. The correlation between the candidate biomarkers’ concentration in milk and the RFI values is shown in Pearson correlation coefficient (r) and *p*-value.

Lactation stage	Metabolites	AUC	CI	r	*p*-value
Early lactation (50 DIM)	C10	0.81	0.67–0.93	0.29	0.070
	Acetylornithine	0.75	0.55–0.89	−0.41	0.009
	SM 16:1	0.75	0.57–0.90	0.38	0.020
	lysoPC a C16:1	0.74	0.57–0.88	0.15	0.350
	lysoPC a C18:1	0.74	0.56–0.88	0.37	0.020
Mid-lactation (150 DIM)	C12:1	0.81	0.64–0.93	−0.48	0.002
	Histidine	0.79	0.63–0.94	0.33	0.040
	Creatinine	0.78	0.61–0.91	0.05	0.740
	C3:1	0.77	0.62–0.91	0.01	0.970
	C16:1-OH	0.75	0.61–0.88	−0.40	0.010
Late lactation (240 DIM)	Phenylalanine	0.85	0.74–0.95	0.39	0.010
	Valine	0.81	0.67–0.93	0.43	0.006
	Methionine	0.79	0.64–0.92	0.39	0.010
	Arginine	0.79	0.64–0.92	0.44	0.005
	Acetylornithine	0.78	0.64–0.92	0.55	<0.001

Correlation analyses were performed between the top five metabolites (ranked according to AUC values) and their corresponding RFI phenotypes at all the three stages of lactation. At the early stage of lactation, C10, SM C16:0, lysoPC a C18:1 were positively correlated (*p* < 0.05) with RFI phenotypes and acetyl-ornithine was negatively correlated (*p* < 0.05) with RFI. At the mid-lactation stage, out of the top five candidate biomarkers, C12:1 and C16:1-OH were negatively (*p* < 0.05) correlated with RFI phenotypes while, histidine had positive correlation (*p* < 0.05) with RFI. All the top five candidate biomarkers at the late stage of lactation are positively correlated (*p* < 0.05) with RFI phenotypes (Table 5).

### Prediction of RFI phenotypes from milk metabolite profiles

A stepwise linear regression analysis was performed to identify milk metabolites that can predict individual RFI phenotypes at early, mid and late lactation stages. Out of the 118 metabolites identified and quantified at each stages of lactation, only metabolites that were significantly different and tended to differ between high and low RFI comparisons were used in the analysis. The analysis was performed on the metabolite concentration data after adjusting for significant fixed effects (parity, MY, HS, and AFC). At the early lactation stage, a total of 24 metabolites were fitted and the model picked 6 metabolites (lysoPC a C18:2, PC aa C40:1, lysoPC a C16:1, acetyl ornithine, citric acid and fumaric acid) as predictors of RFI phenotypes with high accuracy (*R* ^2^ = 0.76, adjusted *R* ^2^ = 0.71, root mean squares of error (RMSE) = 1.64). Leave-one-out cross validation *R* ^2^ (LOOCV *R* ^2^) of 0.65 was observed for the model. Among the 6 metabolites, fumaric acid and lysoPC a C18:2 explained 43% and 34% of the variation explained by the model, respectively. Similarly, at the mid lactation stage, the prediction model was fitted for 28 milk metabolites and the model picked 5 of them (C12.1, PC aa C36:6, C6:1, valine and C12) with relatively lower *R* ^2^ (Model *R* ^2^ = 0.53, adjusted *R* ^2^ = 0.45, RMSE = 2.2 and LOOCV *R* ^2^ = 0.37) compared to the early stage of lactation. A relatively lower prediction accuracy and higher root mean squares of error were observed at the mid lactation stage as compared to the early and late stages. At the late lactation, 37 metabolites were fitted in the model and ornithine, serine, PC ae C40:6, betaine, C0, PC ae 36:0, C6:1 and C16 predicted RFI with relatively higher accuracy (model *R* ^2^ = 0.77, adjusted *R* ^2^ = 0.71, RMSE = 1.73, LOOCV *R* ^2^ = 0.60).

## Discussion

### Lactation stage specific physiological changes in divergent RFI groups

The physiology of residual feed intake during lactation in dairy cows is affected by multi-dimensional factors. The magnitude of change in body reserve mobilization, drainage of nutrients from circulation towards milk synthesis and dry matter intake define the dynamics of physiological state during the lactation period. Taking these dynamics into account, we hypothesized that feed efficient cows may undergo unique physiological adjustments to cope with the metabolic challenges and maintain similar production level from less feed as compared to the feed inefficient cows. To this end, first we compared milk metabolite profiles between high and low RFI cows at the three time points (50, 150, 240 DIM) and traced the pathways enriched by the significantly different metabolite profiles during lactation. Furthermore, we tested the utilization of metabolite profiles which are components or by-products of regulatory networks to identify the feed efficiency level of the cows in the RFI ranking or predict individual RFI phenotypes.

### Elevated acyl carnitines and lysophosphatidylcholines in high RFI cows

The comparison of milk metabolite profiles between high and low RFI cows at the early lactation stage revealed elevated concentrations of medium and long-chain acyl carnitines in high RFI cows. The result is in agreement with previous studies that reported increased plasma concentrations of medium and long chain acyl carnitines as an indication for intracellular energy metabolism pattern and metabolic dysfunction (Koves et al., 2008; Adams et al., 2009; Makrecka-Kuka et al., 2017). Carnitine plays an essential role in energy metabolism with the main function of transferring long-chain fatty acids to mitochondria for subsequent *β*-oxidation (Houten et al., 2016). Incomplete *β*-oxidation gives rise to even chain C4-C22 acylcarnitine species (Koves et al., 2008). The increase in concentration of milk acyl carnitines (C10:2, C10:1, C10, C16:1) in the least efficient cows is likely due to the incomplete fatty acid *β*-oxidation as early lactation is characterized by body fat mobilization to overcome the energy deficit. A study in model animals showed that raising plasma fatty acids induces increased biogenesis of mitochondria in skeletal muscle (Garcia-Roves et al., 2007) and lactating dairy cows divergent in genetic background for milk production had different mitochondrial DNA copy number in liver and mammary gland (Weikard and Kuehn, 2018). These results suggest that low RFI cows might increase their mitochondrial biogenesis in key energetically active tissues (mammary gland, liver and muscle) during early lactation to maximize the oxidation of body fat as compared to the high RFI cows. Conversely, it remains possible that the high RFI cows may have a mismatch between increased level of fat and mitochondrial copy number that may lead to increased levels of medium and long-chain acyl carnitines caused by incomplete *β*-oxidation.

The least efficient cows had increased milk concentration of glycerophospholipids, specifically lysophosphatidylcholines at the early stage of lactation. Lysophosphatidylcholines are formed by hydrolysis of phosphatidylcholines by lipoprotein-associated phospholipase A2 and have been identified as a group of proinflammatory lipids (Schmitz and Ruebsaamen, 2010). The overproduction of lysophosphatidylcholines can result from the overexpression or enhanced activity of enzymes such as lipoprotein-associated phospholipase A2 (Lp-PLA2) in circulation (Thompson et al., 2010). In hepatocytes, higher concentrations of lysophosphocholine disrupts mitochondrial integrity and enhances cytochrome C release (Hollie et al., 2014). The increased milk lysophosphatidylcholine (lysoPC a C20:4, lysoPC a C18:1, lysoPC a C18:2, lysoPC a C16:1, lysoPC a C18:0 and lysoPC a C28:1) concentration in the high RFI group might be due to increased level of lipoprotein-associated phospholipase A2. This may have caused disruption of mitochondrial integrity leading to compromised energy production in the high RFI group. Acyl carnitines and lysophosphatidylcholines are known to activate proinflammatory pathways in rodent models (Hung et al., 2012; Rutkowsky et al., 2014). The energy cost of activating the immune system has been reported to be 0.64 g of glucose/kg of metabolic body weight per hour in dairy cows (Kvidera et al., 2017). Therefore, the increased concentration of acyl carnitines and lysophosphatidylcholines in high RFI cows may have activated the immune system and caused comparatively higher energy loss.

Pathway enrichment analysis for the metabolites that differed (*p* < 0.05) in concentration between least and most efficient cows at the early stage of lactation revealed significant enrichment of TCA cycle and glycerophospholipid metabolism. This indicates that the difference between the least and most efficient lactating cows in energy efficiency at early lactation is likely related to the ATP production efficiency. Of note, mitochondria are responsible for producing over 90% of cellular ATP from acetyl CoA, which is generated upon digestion and catabolism of carbohydrates, protein and lipid derived from the diet and/or body reserves (Cantalapiedra-Hijar et al., 2018). Regulation of the TCA cycle occurs at the points that involve citrate synthase, isocitrate dehydrogenase, and alpha-ketoglutarate dehydrogenase (Cavalcanti et al., 2014). The increased concentration of citric acid in low RFI groups suggests a lower rate of energy production in low RFI cows as glycolysis is inhibited by increased citrate (Wiegand and Remington, 1986).

### Elevated short-chain acyl carnitines and decreased amino acids in low RFI cows

At mid lactation stage short-chain acyl carnitines such as C3 and C5 were elevated in low RFI cows and the concentrations of valine, methionine, histidine and tryptophan were decreased (*p* < 0.05). The reciprocal regulation of short-chain acylcarnitines and valine observed in low RFI cows suggests that low RFI cows may supplement their energy source from valine catabolism compared with the high RFI cows. Amino acid catabolism is a source for C3 and C5 species (Koves et al., 2008) and specifically, valine catabolism results in C3-acylcarnitine production (Newgard, 2012). The oxidation of branched-chain amino acids produces more energy than complete oxidation of glucose in the form of ATP (Monirujjaman and Ferdouse, 2014). The low RFI cows at the mid lactation stage may maintain a comparative energy efficiency by catabolizing amino acids for energy source and subsequently diluting amino acid concentration in milk. Contrary to the early lactation stage, low RFI cows at mid lactation had elevated long-chain acylcarnitines suggesting that low RFI cows oxidize body fat to a certain level as opposed to their high RFI counter parts that may have switched from mobilizing body fat for energy source. In addition, the concentration of methionine was concomitantly increased with the concentration of PC aa C38:6, PC aa C32:2, PC aa C36:6, PC ae C36:0 in high RFI cows and agrees with the finding that methionine activates phosphatidylcholine synthesis (Yao and Vance, 1988).

Compared to high RFI cows, the concentration of the methionine, histidine and tryptophan were decreased (*p* < 0.05) whereas phenylalanine and arginine tended to decrease in low RFI cows. Bifari and Nisoli (2017) reported that the effect of essential amino acids drastically changes when the animal is in catabolic or anabolic condition. In catabolic state, essential amino acids serve as energy substrates while, in anabolic condition they induce protein synthesis and cell growth. The decreased concentrations of these amino acids in milk from low RFI cows is likely due to catabolism that resulted in their subsequent depletion in milk. Notably, histidine, valine and methionine are reported to induce milk protein synthesis in mammary gland epithelial cells *via* the mTOR signalling pathway (Gao et al., 2015; Zhou et al., 2018). Protein production is a costly process and central to the cell physiology (Kafri et al., 2016). In line with this, Aoyagi et al. (1988) estimated the requirement of 7.52 ATPs per peptide synthesis in chicken. Thus, the elevated concentration of these amino acids in high RFI cows suggests that the energy demanding cellular process of protein synthesis or turnover is comparatively higher in high RFI cows. Conversely, the decreased concentration of valine, one of the three branched-chain amino acids, in the low RFI cows suggests that oxidation of amino acids as a fuel source may be taking place and this partly explains the comparative energy efficiency of lactating dairy cows.

### Elevated concentrations of amino acids in high RFI cows at late lactation stage

Concentrations of valine, lysine, histidine, tryptophan, proline, citrulline, ornithine, argenine, phenylalanine, serine, methionine, alanine and glutamine were universally elevated in high RFI cows compared to the low RFI group at 240 DIM. In lactating dairy cows, the mammary gland may contribute 40%–45% of whole body protein flux (Lobley, 2003). Capuco et al. (2001) reported decrease in milk production and quantity of mammary gland epithelial cells by 23% and 17% between 90 and 240 DIM, respectively. The increase in the concentration of milk amino acids in the high RFI group at 240 DIM suggests that high RFI cows partition a higher amount of energy to maintain comparatively elevated levels of amino acid concentrations in milk. Richardson and Herd (2004) showed that 37% of the variation in RFI was explained by protein turnover and Cantalapiedra-Hijar et al. (2018) proposed lower energy metabolic rate that may be caused by decreased protein turnover for low RFI animals. The protein turnover in high RFI cows may be characterized by increased protein synthesis and decreased protein degradation, while the low RFI cows may be characterized by decreased protein synthesis and increased protein degradation. Furthermore, serum methylhistidine concentration is used as indicator of muscle breakdown in dairy cows (Houweling et al., 2012). Similarly, the increased concentration of methylhistidine in high RFI cows observed in our study may suggest increased mammary tissue regression in high RFI cows. Therefore, it is likely that at the late lactation stage the difference in feed efficiency levels at least partly accounted by the decreased protein turnover in low RFI cows.

In addition, the concentration of pyruvic acid was increased in the high RFI group compared to the low RFI cows suggesting lower uptake of pyruvate by mitochondria to be oxidized to acetylCoA as the energy source may have shifted to amino acid catabolism. Branched chain amino acids (BCAA) such as valine are oxidized in peripheral tissue (Monirujjaman and Ferdouse, 2014) and the catabolism of the BCAA in the mammary gland increases significantly during lactation (Manjarin et al., 2014). The proportion of mammary intracellular valine utilized for metabolism other than protein synthesis was 34% and this proportion appeared to remain unaffected by dietary AA regime, indicating that valine may participate considerably in metabolism. In the current study, the concentration of valine in milk from high RFI cows markedly exceeds the concentration in the low RFI groups (12.0 ± 2.8 vs. 3.4 ± 0.8) indicating that low RFI cows obtain more energy by catabolism of valine. The oxidation of BCCA produces more energy than complete oxidation of glucose in the form of ATP (Monirujjaman and Ferdouse, 2014) and this partly explains the comparative energy efficiency of most efficient cows.

### Milk metabolite profiles as predictors of RFI

The result of ROC analysis revealed that decanoylcarnitine (AUC = 0.81) can distinguish high and low RFI cows and is positively correlated with RFI phenotypes at early lactation stage. This indicates that decanoylcarnitine (C10) can be used as a candidate biomarker of RFI with moderate utility and agrees with the finding that accumulation of medium-chain acylcarnitine fatty acid derivatives are markers of incomplete long-chain fatty acid oxidation (Adams et al., 2009). More interestingly, a set of six metabolites predicted individual RFI phenotypes with moderate accuracy and among the 6 metabolites TCA cycle intermediates (fumaric acid and citric acid) and lysoPC a C18:2 accounted 89% of the variation explained by the model. This complements the pathway enrichment analysis that revealed TCA cycle and glycerophopholipid metabolism as the most important pathways enriched at early lactation. At the mid lactation stage, dodecanoycarnitine (C12) was a promising candidate biomarker (AUC = 0.81) of feed efficiency, however, the panels of metabolites identified to predict individual RFI phenotypes had lower prediction potential indicating their more limited applicability. Ultimately, phenylalanine (AUC = 0.85) and valine (AUC = 0.81) were the top 2 candidate biomarkers at late lactation stage. The panels of metabolites identified to predict individual RFI phenotypes showed comparable prediction potential in early lactation. Overall, early lactation is the optimum time period to predict RFI phenotypes from milk metabolite since this time point has higher prediction potential and provides individual RFI estimates earlier during the lactation period which can assist management decisions.

## Conclusion

This study identified lactation stage specific metabolic differences between high and low RFI cows. We utilized these metabolic differences and identified candidate biomarkers that distinguish RFI categories and developed models that can be used to predict RFI phenotypes from panels of milk metabolite profiles. This result has potential for application to improve feed efficiency of dairy cows and reduce the carbon footprint of milk production.

## Data Availability

The original contributions presented in the study are included in the article/supplementary material, further inquiries can be directed to the corresponding author.
